# Deep Learning-Based Human Activity Recognition for Continuous Activity and Gesture Monitoring for Schizophrenia Patients With Negative Symptoms

**DOI:** 10.3389/fpsyt.2020.574375

**Published:** 2020-09-16

**Authors:** Daniel Umbricht, Wei-Yi Cheng, Florian Lipsmeier, Atieh Bamdadian, Michael Lindemann

**Affiliations:** ^1^ Roche Innovation Center Basel, F. Hoffmann-La Roche Ltd, Basel, Switzerland; ^2^ Roche Innovation Center New York, Roche TCRC Inc, NY, United States

**Keywords:** body-worn sensor, digital endpoints, digital health technology, digital outcome measures, gesture detection, human activity recognition, negative symptoms, schizophrenia

## Abstract

**Background:**

We aimed to develop a Human Activity Recognition (HAR) model using a wrist-worn device to assess patient activity in relation to negative symptoms of schizophrenia.

**Methods:**

Data were analyzed in a randomized, three-way cross-over, proof-of-mechanism study (ClinicalTrials.gov: NCT02824055) comparing two doses of RG7203 with placebo, given as adjunct to stable antipsychotic treatment in patients with chronic schizophrenia and moderate levels of negative symptoms. Baseline negative symptoms were assessed using the Positive and Negative Syndrome Scale (PANSS) and Brief Negative Symptom Scale (BNSS). Patients were given a GeneActiv^™^ wrist-worn actigraphy device to wear over a 15-week period. For this analysis, actigraphy data and behavioral and clinical assessments obtained during placebo treatment were used. Motivated behavior was evaluated with a computerized effort-choice task. A trained HAR model was used to classify activity and an activity–time ratio was derived. Gesture events and features were inferred from the HAR-detected activities and the acceleration signal.

**Results:**

Thirty-three patients were enrolled: mean (±SD) age 36.6 ± 7 years; mean (±SD) baseline PANSS negative symptom factor score 23.0 ± 3.5; and mean (±SD) baseline BNSS total score 36.0 ± 11.5. Activity data were collected for 31 patients with a median monitoring time of 1,859 h per patient, equating to ~11 weeks or 74% monitoring ratio. The trained HAR model demonstrated >95% accuracy in separating ambulatory and stationary activities. A positive correlation was seen between the activity–time ratio and the percent of high-effort choices (Spearman r = 0.58; *P* = 0.002) in the effort-choice task. Median daily gesture counts correlated negatively with the BNSS total score (Spearman r = −0.44; *P* = 0.03), specifically with the diminished expression sub-score (Spearman r = −0.42; *P* = 0.03). Gesture features also correlated negatively with the BNSS total score and diminished expression sub-scores. Activity measures showed similar correlations with PANSS negative symptom factor but did not reach significance.

**Conclusion:**

Our findings support the use of wrist-worn devices to derive activity and gesture-based digital outcome measures for patients with schizophrenia with negative symptoms in a clinical trial setting.

## Introduction

Negative symptoms are a key psychopathologic dimension and an important driver of functional disability in schizophrenia with up to 60–70% of patients exhibiting at least one such symptom (1, 2). Despite the high unmet medical need, there is currently no approved treatment for negative symptoms of schizophrenia in the United States.

Factor analyses of negative symptoms have demonstrated at least two dimensions: one consisting of apathy, amotivation, avolition, asociality, and anhedonia (referred to as ‘avolition’); and expressive deficits (including affective flattening and poverty of speech and diminished use of gestures). The first dimension has been shown to be a key driver of functional impairment (3).

Currently, negative symptoms of schizophrenia are primarily assessed with clinician administered rating scales (4). Known problems with rating scales include challenges in establishing interrater reliability in large multinational studies, reliance on patients’ reports for symptoms that are not directly observable in the interview, and expectation bias. These factors reduce the likelihood of signal detection in clinical trials of novel therapies for schizophrenia, increase the risk and cost of drug development, and diminish the chance of finding a treatment for this debilitating disease (5).

The development of alternative methods to objectively assess negative symptoms in patients with schizophrenia, in particular avolition as a key dimension, is critical to further drug development in this disease. Continuous assessment of a patient’s activity by actigraphy may offer such an assessment that provides not only objective, but also longitudinal, data usually not available directly to the clinician. Previously, correlations between actigraphic measures and clinical symptomatology have been reported in patients with schizophrenia. They have shown stability both within and between psychotic episodes (6) and have also been linked to neuroimaging markers (7, 8). In general, reduced activity has been found to correlate with higher negative symptoms, in particular apathy, but not expressive deficits. Although a few studies that obtained symptom assessment and actigraphy measures did not report any correlation, indicating that such correlations were not always found (9–12). However, to our knowledge, all studies used activity counts as the primary variable assessing activity levels. No attempts have been made to differentiate the activity signal into different kinds of activities, in particular into ambulatory and stationary activity (like gesturing while standing or sitting).

Recent developments in digital health technology, including wearable sensors, provide new opportunities to continuously and passively monitor patients over a longer duration of time, collecting rich data sets. Previous studies have found that the use of wearable devices in schizophrenia is feasible and acceptable for patients and may be used to assess heart rate, electrodermal activity, and movement in everyday life (13). Human Activity Recognition (HAR) can identify actions carried out by an individual using acceleration and gyroscope data obtained from various sources including body-worn sensors (14–17).

Here, we present data exploring the use of a wrist-worn device to assess patient activity over a 15-week period to determine how activity measures extracted from the device correlate with negative symptoms of schizophrenia. The key goal was to test new analytical approaches that allow a more fine grained classification of activities in the context of a multi-site clinical trial. In addition, we explored if and how results of this analysis relate to clinical symptoms and, importantly, to performance in the effort-choice task, a behavioral assay probing the reward system and motivated behavior. A relatively large number of studies using effort-choice paradigms have shown that negative symptoms, particularly avolition, are associated with reduced motivation or willingness to expend high efforts for highly rewarded outcomes in such tasks (18–20).

## Materials and Methods

### Study Design

The data were collected in a randomized, three-way cross-over, proof-of-mechanism study conducted between June 28, 2016 and April 28, 2017 (ClinicalTrials.gov: NCT02824055; protocol BP29904). The study compared two doses of the phosphodiesterase-10 inhibitor RG7203 (5 mg and 15 mg) with placebo, given as adjunctive to stable antipsychotic treatment in patients with chronic schizophrenia and moderate levels of negative symptoms. Outpatients were recruited through referral, direct contacts, and advertisements.

Patients were randomized to one of six treatment sequences using a central randomization system. Patients received once-daily placebo, 5 mg RG7203, or 15 mg RG7203 (matching oral capsules). To reach the 15 mg dose, treatment was up-titrated during Week 1. Each treatment period lasted for 3 weeks, followed by a 2-week washout period. For activity monitoring, study participants were provided with a GeneActiv^™^ (Activinsights Ltd, Cambridge, UK) wrist-worn actigraphy device to record data. Patients were asked to wear the device for 24 h each day throughout the entire 15-week trial period. The study was conducted in accordance with the principles of the Declaration of Helsinki and Good Clinical Practice guidelines. All patients provided written informed consent for study participation.

Primary results from the study will be published separately (Umbricht et al. Manuscript submitted for publication). The current analysis did not determine drug efficacy, but leveraged the placebo periods of the study.

### Participants

Eligible patients were aged 18–50 years with a Diagnostic and Statistical Manual of Mental Disorders-5 diagnosis of schizophrenia and a Positive and Negative Syndrome Scale (PANSS) negative symptom factor score (NSFS) ≥18 (21) at screening. Patients were to be symptomatically stable and receiving antipsychotic treatment not exceeding a dose equivalent to 6 mg risperidone. Additional requirements for symptom severity at screening included: a Clinical Global Impression-Severity (CGI-S) score ≥3 (at least mildly ill); a score ≤4 (moderate or less) for PANSS items of hostility (P7) and uncooperativeness (G8); a PANSS depression score (G6) ≤4 (moderate or less); and a score ≤8 on the Calgary Depression Scale for Schizophrenia.

Exclusion criteria included: patients with a score >2 (mild) for any of the four CGI-S items of the Extrapyramidal Symptom (EPS) Rating Scale; electroconvulsive treatment within 6 months of screening, and olanzapine or clozapine within 3 months of screening; use of more than one antidepressant, or a change in dose of antidepressant within 4 weeks of screening; strong/moderate inhibitor or inducer of cytochrome P350 (CYP) 3A or CYPC8 within 14 days of screening; presence of a substance use disorder; positive urine screen for amphetamines, methamphetamines, opiates, buprenorphine, methadone, cannabinoids, cocaine, or barbiturates; a movement disorder that might affect ratings on the EPS scale; or prior or current medical conditions that could impair cognition or psychiatric function.

### Clinical Assessments

Patients were assessed during inperson study visits. Baseline negative symptoms were assessed using the PANSS and Brief Negative Symptom Scale (BNSS) (22, 23). For correlational analysis, the PANSS NSFS, PANSS positive symptom factor score (PSFS) (21), the BNSS total score, and BNSS apathy index and expressive deficits factors (24) were used.

Motivated behavior was assessed with a computerized effort-choice task where patients were given a choice of an easy task with a lower reward or a more difficult task with a higher reward (25). Patients had the option to press a blue balloon 20 times until it popped for which they would receive one point, or to press a green balloon 100, 120, or 150 times until it popped to receive three, five, or seven points. The patients were informed about the maximum possible reward and the probability of receiving the reward (50 or 100%). Each set of cumulated 20 points convert to a $1 bonus. The percentage of high-effort choices across all effort levels and reward levels of five and seven points at 100% probability of receiving the reward was measured to determine the participant’s motivated behavior.

The GeneActiv^™^ actigraphy device recorded the acceleration of wrist movement at 20 Hz to assess patient activity throughout the trial period. For this analysis, actigraphy data and behavioral and clinical assessments obtained during the placebo treatment period were used. The monitoring ratio was calculated by dividing the total number of hours with sensor data collected by the total number of hours in the study.

### Sensor-Based Features

A 9-layer convolutional recurrent neural network (26) was trained using two public annotated data sets [Reiss et al. (27) and Stisen et al. (28)] containing wrist-worn acceleration data for nine subjects each to infer patients’ activities. Performance evaluation of the trained HAR model was conducted using held-out testing data and internally collected sensor data from patients with multiple sclerosis who performed balance tests (stationary) and 2-min walking tests (ambulatory) (29). Two subjects from each of the Reiss and Stisen data sets were left out during training, while sensor data from 14 subjects were used as input to train an activity recognition model. One held-out subject from each data set formed the validation set, which was used to tune the hyper-parameters and determine convergence of the model during training, and one held-out subject from each data set formed the testing set, which was used only for final performance evaluation.

The trained neural network was used to infer a classification of patient activity collected on the actigraphy devices. Patient activity was categorized as either ambulatory (*i.e.* walking, climbing stairs, cycling, jogging) or stationary (*i.e.* sitting, standing, lying down, doing hand work). An activity ratio was derived for each patient based on the activities determined using the HAR and was defined as the total active time involving gait (*i.e.* walking, climbing stairs, running, cycling) divided by the total monitoring time.

Gesture events were inferred from the HAR model-predicted activities, combined with the standard deviation (SD) of the magnitude of acceleration signal from the wrist, using a 0.01 g threshold within a 1-s moving window, inspired by a previously published method by Rai et al. (30). In Rai et al. the authors observed that when the SD of the magnitude of the accelerometer signal was below 0.01 g, the user was idle with 99% probability. Therefore, a gesture event was defined as the time when the patient was not moving (*i.e.* sitting, standing, lying down, doing hand work) according to the HAR model, while the SD of the acceleration signal was >0.01 g. As gesture events were identified by a 1-s moving window across the accelerometer signal, the start and end time of a gesture event was defined by the continuous moving windows that fit the SD criteria, with a maximal gap between eligible windows smaller than 1 s. From the defined gesture events, we calculated gesture count and gesture power. Gesture count was calculated as the total number of gesture events per day. Gesture power was calculated during the detected gesture events by integrating the squared magnitude of the acceleration signal: $\Sigma_{t}\;{\left(m_{t}-\bar{m}\right)}^{2}$, where *m_t_* is the magnitude of accelerometer signal at time *t*, and $\bar{m}$ is the mean of magnitude across time in a gesture event.

### Statistical Analysis

Correlations between clinical scores and sensor-based features were evaluated using Spearman’s correlation coefficient.

## Results

### Study Participants

In total, 33 patients with negative symptoms of schizophrenia were enrolled at three study centers in the United States. Study participants had a mean (±SD) age of 36.6 ± 7 years, and the majority were male (30/33) and Black (21/33) (**Table 1**). The mean (±SD) baseline PANSS NSFS was 23.0 ± 3.5, the mean (± SD) baseline BNSS total score 36.0 ± 11.5, and the mean CGI-S score 3.7 ± 0.5.

**Table 1:** Demographics and clinical characteristics at screening.

	Cohort (N = 33)
Mean age ± SD (years)	36.6 ± 7.0
Male gender, n (%)	30 (91)
Race, n (%) Black White Asian	21 (64) 9 (27) 3 (9)
Mean BNSS total score ± SD	36.0 ± 11.5
Mean PANSS NSFS ± SD	23.0 ± 3.5
Mean PANSS PSFS ± SD	19.2 ± 4.8

BNSS, Brief Negative Symptom Scale; NSFS, Negative Symptom Factor Score; PANSS, Positive and Negative Syndrome Scale; PSFS, Positive Symptom Factor Score; SD, standard deviation.

### Activity Data

Overall, 31 patients agreed to wear a GeneActiv^™^ wrist-worn actigraphy device to record actigraphy data. Median collected monitoring data per patient was 1,859 h equating to around 11 weeks or 74% monitoring ratio. There was no significant correlation between baseline PANSS NSFS, PANSS PSFS, or BNSS total score and monitoring ratio.

### Validation of Human Activity Recognition Model

Based on the held-out validation data, the trained HAR model demonstrated >95% accuracy in separating ambulatory (*i.e.* walking, climbing stairs, cycling, jogging) and stationary activities (*i.e.* sitting, standing, lying down, doing hand work). The model showed 94.9 and 95.5% accuracy in identifying stationary and ambulatory activities, respectively.

### Correlation of Actigraphy-Derived Features With Clinical Scores

**Figure 1:**
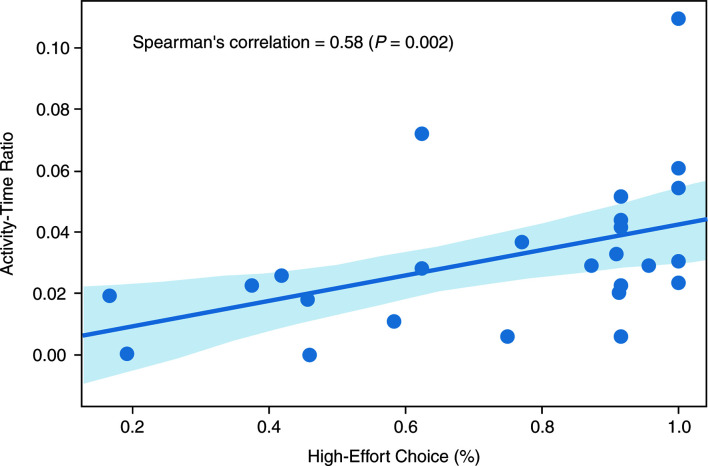
Spearman’s correlation between activity–time ratio and high-effort choice (Spearman’s r = 0.58; *P* = 0.002).

The activity–time ratio correlated positively with the percent of high-effort choices at the end of the placebo period (Spearman’s r = 0.58; *P* = 0.002; **Figure 1**).

**Figure 2:**
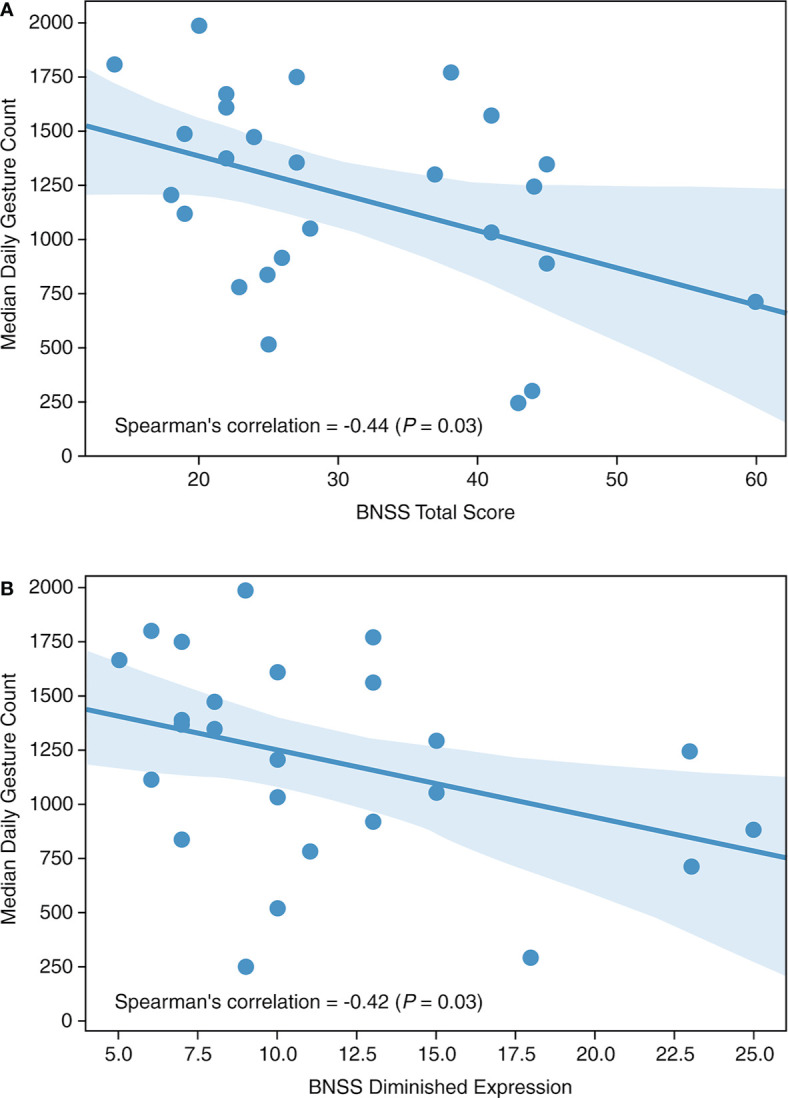
Median daily gesture count *versus* **(A)** BNSS total score and **(B)** BNSS diminished expression. BNSS, Brief Negative Symptom Scale.

Median daily gesture counts were negatively correlated with the BNSS total score (Spearman’s r = −0.44; *P* = 0.03; **Figure 2A**) at the end of the placebo period, specifically with the diminished expression sub-score (Spearman’s r = −0.42; *P* = 0.03; **Figure 2B**). No correlation was observed with the BNSS apathy sub-score or the PANSS NSFS. Gesture power and activity–time ratio correlated negatively with the diminished expression sub-score, but not with other measures of negative symptoms (**Table 2**). Notably, all significant correlations were in the *a priori* expected direction supporting the convergent validity of the proposed novel digital measures with the established clinical scales.

**Table 2:** Spearman’s correlation between high-effort choice, activity, and gesture features with clinical scores.

	High-effort choice in effort-choice task (N = 27)	Activity ratio (N = 26)	Gesture power (N = 26)	Gesture count (N = 26)
BNSS Apathy Index	0.12	−0.025	−0.178	−0.277
BNSS Diminished Expression	0.06	−0.210	−0.423*	−0.424*
BNSS Total Score	0.12	−0.080	−0.312	−0.438*
PANSS NSFS	0.02	−0.256	0.080	−0.251
PANSS PSFS	−	−0.149	0.164	−0.026

*P < 0.05. Higher BNSS scores indicate greater disease severity; cells showing significant correlations are shaded in gray.

BNSS, Brief Negative Symptom Scale; NSFS, Negative Symptom Factor Score; PANSS, Positive and Negative Syndrome Scale; PSFS, Positive Symptom Factor Score.

Performance in the effort-choice task did not correlate with any clinical measure of negative symptoms.

## Discussion

The results of this analysis demonstrate that the use of a wrist-worn actigraphy device is feasible to support continuous monitoring of clinically relevant behavior in a multi-site clinical trial setting over an extended period of time. The patient monitoring ratio was acceptable throughout the trial, allowing for a high volume of data to be collected by the end of the placebo period. This suggests that patients were comfortable wearing the device and that the device is suitable for continuous assessment over a number of weeks.

Previous studies have investigated the feasibility and use of wearable devices in schizophrenia (10, 31). Cella et al. examined the use of a novel mobile health method using wearable technology to determine illness severity in patients with schizophrenia. The device was acceptable to patients and provided accurate and reliable measures of everyday activity and behavior, including assessment of physiological measures, functioning symptoms, and levels of medication (13). Meyer et al. utilized a combination of wrist-worn devices and smartphones to continuously monitor sleep and rest-activity profiles in people with schizophrenia over a 2-month period. All study participants exceeded the 70% threshold for feasibility of the wearable device with a mean wear time of 21.8 h per day or 91% of the total study duration (31).

Passive monitoring has also been used to quantify behavioral changes in several neurological conditions, including Parkinson’s disease (32, 33), multiple sclerosis (29), and Huntington’s disease (34, 35). Lipsmeier et al. assessed the feasibility, reliability, and validity of smartphone-based digital biomarkers of Parkinson’s disease over a 6-month period in a phase I clinical trial (32). Adherence was acceptable and sensor-based features showed moderate to excellent test–retest reliability (32). The use of smartphone- and smartwatch-based remote patient monitoring was also employed by Midaglia et al. in a 24-week pilot study in patients with multiple sclerosis (29). Adherence to passive monitoring was 70.8% with patient satisfaction rated as good to excellent, which remained stable throughout the study (29). An ongoing Digital-HD study is investigating the tolerability and feasibility of smartphone-based technology to passively monitor motor and non-motor manifestations of Huntington’s disease (35). These studies indicate the potential for the measurement of disease-relevant features from daily life in a clinical trial setting.

While most, if not all, previous actigraphy studies in schizophrenia have used simple activity counts as a measure of patient activity, our approach used machine learning to extend beyond a simple count, to identification specifically of gesture events during non-locomotive activities and hence, eliminates the situation where hand movements are caused by walking or running. Our HAR model was validated using previously published data, with a high level of accuracy, demonstrating that this model is reliable and robust for the detection of ambulatory *versus* stationary activity. There was a significant positive correlation (*P* = 0.002) between the activity–time ratio and the percent of high-effort choices, indicating an association of avolition and lower activity in daily life. As expected, gesture features derived from the HAR model were associated with expressive deficits, supporting the validity of activity and gesture-based digital outcome measures for negative symptoms in patients with schizophrenia.

Our study also highlights well-known problems with clinician administered rating scales. We did not find correlations between percentage of high effort-choice in the effort-choice task and the various clinical assessments of negative symptoms, in particular apathy, as previously reported by others (12). This may be due to differences in the patient sample. However, the correlations between the effort-choice performance and the activity index would indicate that both measures indeed capture an aspect of reduced motivation. The lack of an association between the clinical assessments of apathy and the effort-choice performance thus suggests that the clinical assessments capture apathy unreliably, which may be the key reason for the lack of association between activity measures and apathy. This is not surprising, as the key features of apathy cannot be observed in the interview but have to be elicited by the clinician and rely primarily on the patient’s memory and report, which has been shown to negatively affect the accuracy of symptom reporting (36). Not surprisingly, we found the highest correlation between the expressive deficit scores and gesture count and power. Expressive deficits are directly observable in the interview and hence can be assessed more reliably.

Our study has several limitations. Firstly, we could not validate our findings in a larger cohort of patients. This will be a critical step in establishing our analytical approach as a tool to assess negative symptoms. Secondly, our study did not allow the establishment of test–retest reliability, *i.e.*, to establish the stability of these measures in stable patients. Both issues are key for implementation of these measures in future clinical trials. Also, our approach focused on stationary periods and did not include gesture events during non-stationary activity which of course occur as well. Identification and inclusion of these additional gesture events should be attempted in future studies and may increase the correlation with negative symptoms. Also, it is possible that among events counted as gestures some may have been included that comprised movements that did not represent gestures such as playing instruments, doing crafts, cooking, other household chores. Excluding such activities in future studies may increase the sensitivity of our approach. In addition, although our approach is a step forward in differentiating gestures from non-gesture activity, it does not allow the differentiation of communicative and socially relevant gestures and gestures that do not have such characteristics. It would require the establishment of an ‘alphabet’ of such gestures in healthy volunteers in terms of actigraphy features that could then be used to detect the presence or absence of communicative gestures in patients—a relevant aspect for the assessment of negative symptoms. Also, previous studies have investigated the use, perception and imitation of gestures by patients in much more detail by direct observation or video-based studies (37, 38) and found abnormalities in all three aspects. Obviously a passive monitoring system like actigraphy is not able to provide these kinds of data. Finally, differences in publicly available data sets of healthy volunteers that were used for validation, including the method of detection, of activities measured, was also a limitation. We did not have a comparison sample of healthy volunteers in whom data were obtained with the same device. It is also conceivable that the very nature of gestures differs between healthy volunteers and patients; however, we are not aware of evidence supporting this assumption. Furthermore, the HAR model has not yet been tested in a drug-based clinical trial or in patient subgroups with baseline characteristics other than negative symptoms.

## Conclusions

Overall, our findings support the use of wrist-worn devices to derive activity and gesture-based digital outcome measures for patients with schizophrenia with negative symptoms in a clinical trial setting. We present initial evidence of convergent validity of sensor-based features with established clinical outcome measures. This could, in the future, enable the objective measurement of behavioral changes in schizophrenia and pave the way towards novel ways to evaluate treatments for the negative symptoms of schizophrenia, thereby supporting essential drug development for these patients.

## Prior Presentation of the Data

Umbricht D, Cheng W-Y, Lipsmeier F, Bamdadian A, Tamburri P, Lindemann M. Deep learning-based human activity recognition for continuous activity and gesture monitoring for schizophrenia patients with negative symptoms. ACNP 57th Annual Meeting 2018. Abstract T210.

## Data Availability Statement

Qualified researchers may request access to individual patient level data through the clinical study data request platform (https://vivli.org/). Further details on Roche’s criteria for eligible studies are available here (https://vivli.org/members/ourmembers/). For further details on Roche’s Global Policy on the Sharing of Clinical Information and how to request access to related clinical study documents, see here (https://www.roche.com/research_and_development/who_we_are_how_we_work/clinical_trials/our_commitment_to_data_sharing.htm).

## Ethics Statement

The studies involving human participants were reviewed and approved by Copernicus Group IRB, PO Box 110605, Research Triangle Park, NC 27709; Washington University in St Louis Human Protection Office, 660 South Euclid Ave. Campus Box 8089 St Louis, MO 63110; Alpha IRB, 1001 Avenida Pico, Suite C#497 San Clemente, CA 92673; Integ Review IRB, 3815 S. Capital of Texas Hwy, Suite 320, Austin TX 78704. The patients/participants provided their written informed consent to participate in this study.

## Author Contributions

DU, FL, and ML contributed to the conception and design of the study. W-YC performed the statistical analysis. All authors interpreted the data and commented on the draft manuscript. All authors contributed to the article and approved the submitted version.

## Funding

This work was supported by F. Hoffmann-La Roche Ltd.

## Conflict of Interest

All authors are employed by company F. Hoffmann - La Roche Ltd. 

The remaining authors declare that the research was conducted in the absence of any commercial or financial relationships that could be construed as a potential conflict of interest.
